# A medicolegal study of domestic violence in south region of Jordan

**DOI:** 10.1186/s41935-017-0006-x

**Published:** 2017-07-18

**Authors:** Hasan Al-Hawari, Asmaa El-Banna

**Affiliations:** 1 Directorate of Forensic Medicine, Al-Karak, south region Jordan; 2 Department of Forensic Medicine& Clinical Toxicology, Faculty of Medicine, Alexandria University, Alexandria, Egypt

**Keywords:** Medico-legal, domestic violence, Jordan

## Abstract

**Background:**

Domestic violence is a forced pattern of behavior that happened in domestic settings to gain or maintain power and control over an individual. The aim of this work is to study the magnitude of domestic violence in south region of Jordan.

**Methods:**

The retrospective study was retrieved from the medico-legal reports of domestic violence cases referred to the Directorate of Forensic Medicine in south region of Jordan during six years period starting from 1st of January 2010 till the end of December 2015.

**Results:**

The total number of cases was 128. The majority was females (77.3%), high prevalence (41.4%) was found in adult age group (18 years and above) in both genders. The prevalence was higher in rural areas (75%). Sexual abuse was the commonest method of domestic violence in both genders (41.4%). Nearly a quarter of cases (23.4%) leaving home as an outcome. Spring months represented the highest percentage of domestic violence occurrence (28.1%). Family and financial problems were reported in 71.1% of cases.

**Conclusion:**

Domestic violence rate in South Jordan is much lower than in other areas but still considered a problem and should be given high priority with regard to prevention, investigation and treatment.

## Background

Domestic violence is a human rights crime and a costly public health problem all over the world. No country is immune to the grave effects of violence. Every year, it causes over 1.6 million deaths. Most of the victims are women. Violence is one of causes of mortality and disability all over the world for women of age 15 to 44 years (Haddad et al. 2011).

There is no universally accepted definition of family violence; it can be as “a range of abusive behaviors that happen in domestic settings or within relationships based on intimacy, trust or dependency” (Report 2008). Actually, family relationships have been defined by the accused person’s relationship to the victim through blood, marriage, adoption or foster care (Sinha 2012).

The domestic violence term has a broader meaning than family violence. As, it includes violence perpetrated not only against family members but also nonfamily members in cohabitation with the family, such as domestic workers (Kazarian 2015).

Family violence includes physical, psychological, sexual, financial and emotional abuse and encompasses controlling and coercive behavior, female genital mutilation and honor-based violence (Krug et al. 2002; Guy et al. 2014).

Literatures report a significant discrepancy regarding definition of domestic violence among different cultures (Sokoloff & Dupont 2005; Fernandez 2006). In Russia, for example, domestic violence means “home violence”. In Ghana, domestic violence is the beating of children, while in Chile; it means the private violence which is directed primarily towards women and children. Whereas in Japan, domestic violence is referred to filial or (offspring-related) violence; children’s physical/emotional reactions to family members. In addition, domestic violence in Japan could be the treatment of the daughter-in-law by the mother-in-law or vice versa (Fernandez 2006; Kozu 1999).

Domestic violence was ranked first type among various types of violence (Faramarzi et al. 2005). Child maltreatment commonly accompanies domestic violence and the rates of co-occurrence range from 30% to 60% (Kelleher et al. 2006).

The south region of Jordan includes four governorates (Karak, Tafileh, Ma'an and Aqaba), the percentage of population living in this area constitutes 9.4% of all Jordanian population (626, 200 people) although the south region constitutes 50.45% of total surface area of Jordan (Department of Statistics (DOS 2013; Hunaiti et al. 2014).

The Jordanian social culture accepts the use of violence with children or women as a kind of discipline, and this acceptance is supported by cultural and social norms. (Al-Badayneh 2012; Gharaibeh & Al–Ma’aitah 2002) Okour and Hijazi (2009) (Okour & Hijazi 2009) found that university students were significantly influenced by witnessing and exposure to family violence.

There is a widespread underreporting of domestic violence cases internationally. Estimates of violence within the family context are difficult to obtain due to conventional perceptions, that violence within the family context is not considered violence (Yoshihama 2002). Moreover, the social behavior and norms in Eastern societies state that relationships and family ties are highly important even when the costs of these exceed the benefits (Fernandez 2006).

Violence is a problem that exists in Jordan and its actual size is not known due to lack of documentation and underreporting (Gharaibeh et al. 2008). Without good accurate information, prevention programs cannot be developed. So, the aim of the present work is to study the magnitude of the problem of domestic violence in south region of Jordan as regard prevalence and rate of domestic violence, socio-demographic data of the victims, risk factors, date, types and outcome of violence.

## Subjects and Methods

The research was a retrospective study. The data was retrieved from the medico-legal reports of domestic violence cases referred to the Directorate of Forensic Medicine in south region of Jordan during a period of six years starting from 1st of January 2010 till the end of December 2015. The total number of cases reported to the police as domestic violence cases during the whole period of study was 170. All of them were alive. Factual cases were 128 while the remaining 42 cases were claims known either by presence of fabricated wounds or by victims confession of these false allegations.

The subjects in the present study were categorized into four groups; first group was preschool children, the second group was children between school age (6 years) and below the mean age of puberty (12 years), the third group was those between the mean age of puberty (12 years) and below legal adulthood age, lastly, the fourth group was the adult age group.

All individuals included in the current study were Jordanians. All domestic violence records were thoroughly reviewed for the following information:

- Socio-demographic data of the victim, including age, sex, residence and risk factors like family or financial problems, family disintegration, study or mental problems and work as a servant.
- Date of violence accident, further divided according to the season.
- Type of violence accident.
- Results of forensic examination, including type of physical abuse (whether using blunt or sharp instrument or burn), also the type of sexual abuse (whether rape or sodomy).
- Immediate complications were recorded while delayed outcome of abuse was obtained after a period of follow up in certain situations as cases of rape for fear of pregnancy and cases of major trauma for fear of development of infirmity and the period of follow up differed according to the case.

Official approval was obtained from Directorate of Forensic Medicine of south region, Jordan. Complete confidentiality was ensured all through the study procedure. The study protocol was approved by the ethical committee of Alexandria Faculty of Medicine.

### Statistical analysis

Data collected were organized, tabulated, and statistically analyzed using the SPSS software package version 20 to obtain mean and standard deviation. Student t- test was used for comparison between two means. Chi square test was used to study significant association between two qualitative variables. P value less than 0.05 was considered statistically significant (Kirkpatrick & Feeney 2013).

## Results

### Prevalence and rate of domestic violence cases

The total number of cases was 128 along the six years of the present study. So, the average rate of domestic violence per year was 4.08/100,000 population.

### Age and sex

**Fig. 1:**
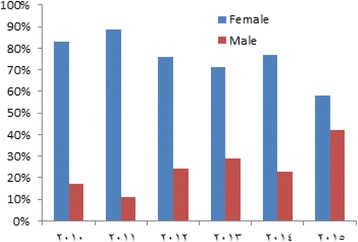
Distribution of domestic violence victims according to gender over the studied years

Table 1 illustrates that the age and sex distribution of domestic violence cases. 77.3% were females (*n* = 99) and 22.7% were males (n = 29), with female to male sex ratio of 3.4: 1. Females outnumbered males in all studied years as shown in Fig. 1. The mean age of cases was 15.85 ± 8.63 years. There was a statistically significant difference between males and females regarding age (P < 0.05). None of cases of elderly abuse was encountered in the present study.

**Table 1:** Age comparison between male and female cases of domestic violence in south region, Jordan (*n* = 128)

	number	%	Range (years)	Mean ± SD	t	*P*-value
Male	29	22.7	41 (5–46)	14.22 ± 10.35	1.982	0.049*
Female	99	77.3	43.5 (1.5 - 45)	17.49 ± 6.92		
Total	128	100	44.5 (1.5-46)	15.85 ± 8.63		

*P*-value was considered significant (*) if ≤ 0.05

Table 2 shows that the highest frequency of domestic violence occurred in age group 18 years and above which accounted for 41.4%, followed by those in age group from 12 to below 18 years. There was a highly statistically significant difference between males and females regarding age groups (p = 0.000*), where females markedly outnumbered males in age groups from 12–18 years and age group 18 years and above.

**Table 2:** Relation between age groups and gender in cases of domestic violence in south region, Jordan (n = 128)

Age group	Sex		Total		X^2^	*P*-value
	Male	Female		%		
<6y	4	4	8	6.25		
6- < 12ys	12	13	25	19.55	18.177	0.000*
12- < 18ys	8	34	42	32.8		
≥18ys	5	48	53	41.4		
Total	29	99	128			

*P*-value was considered significant (*) if ≤ 0.05

#### Residence of victims

the present study revealed that 75% of domestic violence victims reside rural houses while 19.5% of cases reside urban houses. The remaining 5.5% of cases live in shelters.

#### Season of domestic violence

**Fig. 2:**
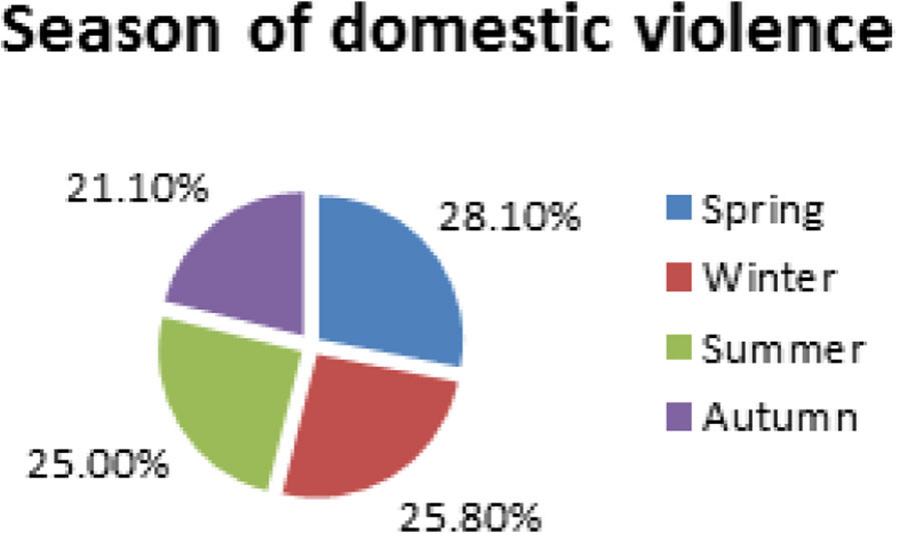
Distribution of studied cases according to seasons of the year

Figure 2 illustrates that the highest percentage of studied cases (28.1%) occurred during the spring months, then they decreased through winter and summer months (25.8% and 25% respectively), to reach 21.1% in autumn months.

#### Risk Factors

**Fig. 3:**
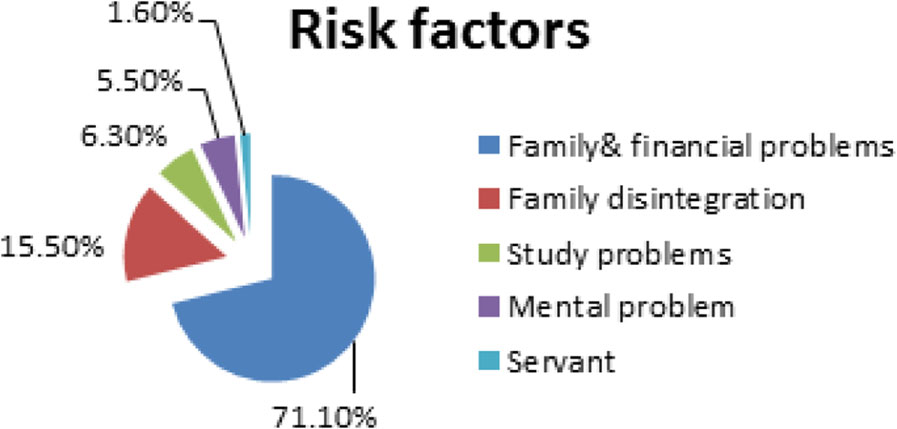
Distribution of studied cases according to risk factors

Figure 3 demonstrates that family and financial problems accounted for the 71.1% of all risk factors of domestic violence, followed by family disintegration (15.5%). Moreover, risk factors related to the victim like studying problems which represented 6.3% and mental problem (5.5%) or being a servant which accounted for 1.6% of cases.

### Type of violence

#### Method of violence

**Fig. 4:**
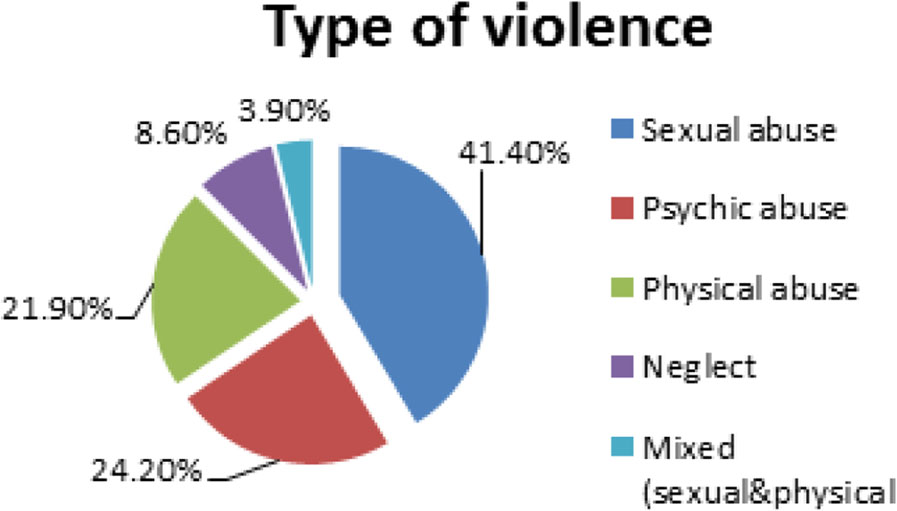
Distribution of studied cases according to type of violence

sexual abuse was the most common method of domestic violence in both genders (41.4%), followed by psychic and physical abuse (24.2%, 21.9% respectively). Then neglect accounted for 8.6% of cases and lastly mixed sexual and physical abuse accounted for 3.9% of cases (Fig. 4).

### Type of violence in relation to age

Table 3 shows that physical abuse was the commonest type of domestic violence committed in the young age groups (below six years and from 6 to 12 years), while the incidence of sexual abuse assaults increased with increasing age, where sexual abuse accounted for the highest percentage of abuse in older age groups (from 12 to 18 years and ≥ 18 years). None of cases of neglect occurred in age group of 18 years and above. There was a highly statistical significant association between age group of the studied cases and the type of violence, where *P* = 0.000*.

**Table 3:** Relation between age groups and type of violence

Age group	Type					Total	X^2^	*P*-value
	Physical	Sexual	Mixed (physical& sexual)	Psychic	Neglect			
<6y	3	1	0	2	2	8		
6- < 12ys	10	5	0	3	7	25	35.151	0.000*
12 < 18ys	5	21	2	12	2	42		
>18ys	10	26	3	14	0	53		
Total	28	53	5	31	11	128		

*P*-value was considered significant (*) if ≤ 0.05

### Type of violence in relation to gender

There was a highly statistical significant relation between gender and the type of domestic violence, where X^2^ = 33.15, P = 0.0000*. Physical abuse represented the commonest type of abuse committed in males (58.6%), moreover, only one case of psychic abuse and none of cases of mixed sexual and physical abuse were reported in males, while sexual abuse was the most frequent type of abuse committed in females (45.4%) followed by psychic abuse (30.3%). The study revealed that females were more than six times as likely as males to be a victim of sexual assault (6.25:1). (Table 4)

**Table 4:** Relation between gender and type of violence among the studied cases

	Type					Total	X^2^	*P*-value
	Physical	Sexual	Mixed(physical& sexual)	Psychic	Neglect			
Male no. %	17	8	0	1	3	29	33.15	0.000*
	58.6%	27.6%	0.00%	3.5%	10.3%	100%		
Female no. = %	11	45	5	30	8	99		
	11.1%	45.4%	5.1%	30.3%	8.1%	100%		
Total no.	28	53	5	31	11	128		
%	21.9%	41.4%	3.9%	24.2%	8.6%	100%		

*P*-value was considered significant (*) if ≤ 0.05

Table 5 shows that there was a highly statistical significant relation between risk factor and the type of domestic violence, where X^2^ = 36.08, P = 0.003*. All mentally retarded cases were abused physically and the two servant cases were sexually abused. Nearly half of cases with family disintegration risk had sexual abuse.

**Table 5:** Relation between risk factor and type of violence among the studied cases

Type	Risk Factor					Total	X^2^	*P*-value
	family &financial problems	Study problems	family disintegration &poverty	servant	MR			
Physical	17	3	1	0	7	28	36.08	0.003*
Sexual	39	3	9	2	0	53		
Mixed (Physical&Sexual)	3	0	2	0	0	5		
Neglect	8	0	3	0	0	11		
Psychic	24	2	5	0	0	31		
Total	91	8	20	2	7	128		

*P*-value was considered significant (*) if ≤ 0.05

### Outcome

**Fig. 5:**
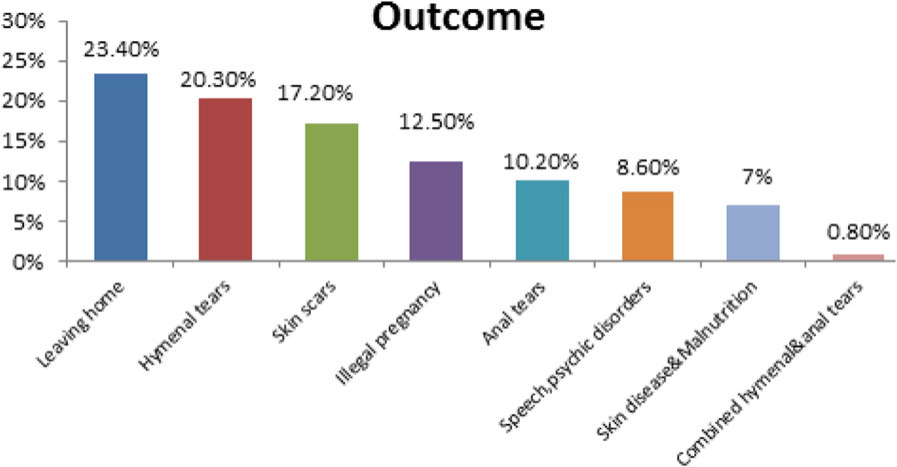
Distribution of studied cases according to outcome

The present study revealed that leaving home was the commonest outcome of domestic violence (23%), followed by occurrence of hymenal tears in sexual abuse cases (20%). Moreover, illegal pregnancy occurred in 12.5% of domestic violence victims. Anal tears accounted for 10% of overall outcome. In addition, a combined hymenal and anal tear occurred in 15.5 years old female victim (Fig. 5).

As regard outcome, Table 6 shows that there was a highly statistical significant relation between age group and outcome, where X^2^ = 58.64, P = 0.0000*. The highest percentage of skin scars (45.5%) was reported among those with age group from 6 to 12 years. On contrary, the highest percentages of hymenal tears and leaving home were recorded in older age group 18 years and above (65.5% and 56.7% respectively).

**Table 6:** Relation between age group and outcome among the studied cases

	Outcome								Total	X^2^	*P*-value
Age (Y)	Skin scars	Hymenal tears	Skin diseases, Malnutrition	Leaving home	Anal Tear	Illegal Pregnancy	Speech, psychic disorders	Combined hymnal & anal tears			
<6No. %	3 13.6%	1 3.8%	2 22.2%	1 3.3%	0 0.0%	0 0.0%	1 9.1%	0 0.0%	8	58.64	0.000*
6- < 12 No. %	10 45.5%	2 7.6%	5 55.6%	0 0.0%	3 23%	0 0.0%	5 45.5%	0 0.0%	25		
12- < 18 No. %	3 13.6%	6 23.1%	2 22.2%	12 40%	5 38.5%	10	3 27.3%	1 100%	42		
>18 No. %	6 27.3%	17 65.5%	00.0%	17 56.7%	5 38.5%	6	2 18.1%	0 0.0%	53		
Total No. %	22 100%	26 100%	9 100%	30 100%	13 100%	16 100%	11 100%	1 100%	128 100%		

*P*-value was considered significant (*) if ≤ 0.05

Table 7 shows that there was a highly statistical significant relation between gender and outcome, where X^2^ = 64.79, P = 0.0000*. The highest percentage of skin scars occurred among males (72.7%), while 81.8% of cases with speech and psychic disorders were females. Moreover, all cases of leaving home were females. Males constituted 61.5% of all cases of sodomy.

**Table 7:** Relation between gender and outcome among the studied cases

Sex	Outcome								Total	X^2^	*P*-value
	Skin scars	Hymenal tears	Skin diseases, Malnutrition	Leaving home	Anal Tear	Illegal Pregnancy	Speech, psychic disorders	combined (hymenal & anal) tears			
Male											
No. %	16 72.7%	0 0.0%	3 33.3%	0 0.0%	8 61.5%	0 0.0%	2 18.2%	0 0.0%	29 22.6%	64.79	0.000*
Female											
No. %	6 29.3%	26 100%	6 66.7%	30 100%	5 38.5%	16 100%	9 81.8%	1 100%	99 77.4%		
Total											
No. %	22 100%	26 100%	9 !00%	30 100%	13 100%	16 100%	11 100%	1 100%	128 100%		

*P*-value was considered significant (*) if ≤ 0.05

Table 8 shows that there was a highly statistical significant relation between type of violence and outcome, where X^2^ = 316.99, P = 0.0000*. It was recorded that 78.5% of physically abused victims developed skin scars and 80.6% of psychic abuse cases leaving their home as an outcome. Regarding sexually abused victims, it was recorded that 41.5% of them had hymenal tears and 30.2% of them had illegal pregnancy.

**Table 8:** Relation between type of violence and outcome among the studied cases

Type	Outcome								Total	X^2^	*P*-value
	Skin scars	Hymenal tears	Skin diseases, Malnutrition	Leaving home	Anal Tear	Illegal Pregnancy	Speech, psychic disorders	combined (hymenal & anal) tears			
Physical	22	0	0	3	0	0	3	0	28	316.99	0.000*
Sexual	0	22	0	2	12	16	0	1	53		
Mixed(Physical &Sexual)	0	4	0	0	1	0	0	0	5		
Neglect	0	0	9	0	0	0	2	0	11		
Psychic	0	0	0	25	0	0	6	0	31		
Total	22	26	9	30	13	16	11	1	128		

*P*-value was considered significant (*) if ≤ 0.05

### Data related to forensic examination

#### Type of physical abuse

**Fig. 6:**
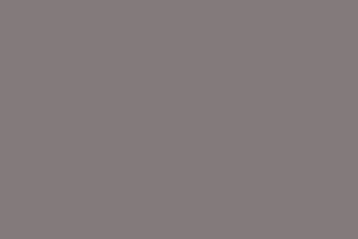
shows contusions on *right* side of face

**Fig. 7:**
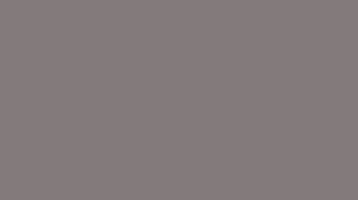
shows contusion in inner side of *upper* lip

**Fig. 8:**
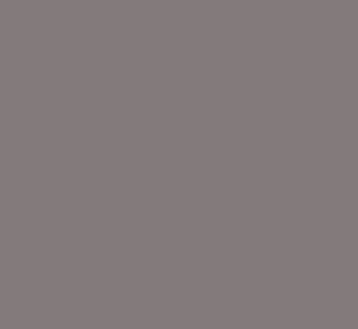
shows multiple abrasions and contusions on a child neck

**Fig. 9:**
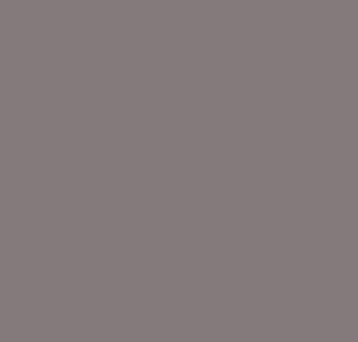
shows multiple cigarette burns on child buttocks

**Fig. 10:**
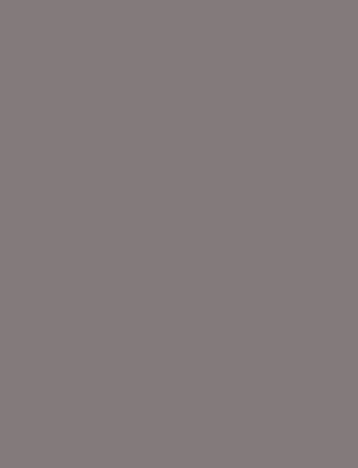
shows multiple cut wounds on child *left* arm

Forensic examination revealed that majority of cases were abused by blunt instruments manifested as patterned abrasions and bruises (71.5%), while seven victims out of 28 cases were abused by intentional burns (25%). Only one case was abused using sharp instrument in Al-Karak shelter for mentally disabled persons (Figs. 6, 7, 8, 9, 10).

#### Type of sexual abuse

**Fig. 11:**
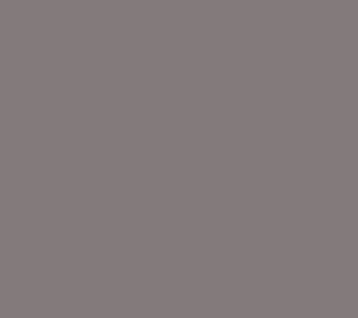
shows recent anal tear in sodomy attack

**Fig. 12:**
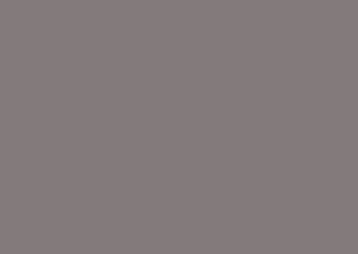
shows multiple suction bruises on a child body

During examination of cases, it was found that 26 cases had recent torn hymen while 16 cases had torn hymen with illegal pregnancy, 12 cases with recent anal tears denoting recent sodomy, while one case showed signs of habitual sodomy. There was also 15.5 years old female victim showed combined abuse (old hymenal tear together with habitual sodomy) (Figs. 11, 12).

## Discussion

In Arab world, domestic violence and abuse is a hidden or masked epidemic. The tragic abuse and neglect of children, spouses, and elderly within the domestic setting is an important cause of long-term problems for families and communities (Kazarian 2015). Domestic violence has inter-generational consequences resulting in repetition of abusive and violent behaviors. Children experienced domestic abuse display increased fear, depression, aggression as well as high levels of antisocial behavior, which can persist into adulthood (Guy et al. 2014).

The total number of cases was 128 along the six years of the present study. So, the average rate of domestic violence per year was 4.08/100,000 population. This is lower than the rate had been recorded about children victims of severe neglect and violence in Amman from 1998 to 2001 which was a rate of 1.2 per 1,000. This was also low if compared to the standard international rate of 4 per 1,000 (Gharaibeh et al. 2007).

This may be explained by the culture of south Jordan that consider notification to authorities is a shameful behavior or may be due to lack of data base, also the use of traditional solving of domestic troubles in Arabian sitting may have a role.

Minimal data is known about violence against females in the Arab society (Btoush & Haj-Yahia 2008) and most literatures on violence against females are confined to the Western societies.

The reluctance of females to report domestic violence and ask for help, can be due to a lot of factors such as lack of available services for victims of violence, the expected harm to the reputation of females and families and the fear from generalized negative stigma attached to the victim of violence and the other family members. In addition to women’s belief that nothing will be changed whatever she did. Asking for help might be considered as a woman revolution against her husband and family, which might cost her a lot of negative familial and social consequences such as divorce (Haj-Yahia 2000; Al-Badayneh 2005).

Since women’s participation in the labor force is minimal, that women’s financial dependence on men, fear of losing their children, a lack of family support, and the social stigma of divorce compels a lot of women to accept and remain in violent relationships (Clark et al. 2012).

Abu Ghazaleh (2008) reported that the main three reasons for hiding the family violence from others are fear of family fragmentation, hazard of having to abandon children in case of divorce, and fear of harming the family’s reputation.

Moreover, the reduced levels of detection of child maltreatment and subsequent reporting may be attributed to inability or reluctance of children to report their victimization due to their small ages and immature stage of development whether physical, mental and cognitive, together with lack of experience and fear of consequences (Kesner et al. 2009).

It is worth noting that none of cases of elderly abuse was recorded in the present work and this indicates the respect of elderly in south region of Jordan.

Regarding to type of violence in relation to age and sex, the distribution of domestic violence cases showed that female to male sex ratio was 3.4: 1 and females outnumbered males in all studied years. There was a statistically significant difference between males and females regarding age (P < 0.05). In consistence, Walker (1999) (Walker 1999) recorded that the single most powerful risk factor for becoming a victim of violence is to be a female.

This finding was consistent with literature on domestic violence in Jordan indicating that females are more likely to be subjected to violence than male members of the family (Araji & Carlson 2001). Similar result was reported in Canada, where 7 in 10 (70%) victims of police-reported family violence were girls or women (Sinha 2012).

Al-Badayneh (2012) (Al-Badayneh 2012) stated that the distribution of power in a Jordanian family is hierarchic, where men are superior while women and children are subordinates. Masculinity in many cultures is associated with power and domination. The father abused everyone, the mother abused her children, then the male children abused the female children. Furthermore, honor crimes, early marriages, gender inequality and deprivation of right to inheritance are all practiced in Jordanian society (Darwazeh 2008).

Studies in developing countries recorded high rates of acceptance of wife beating, reaching up to 69% of Jordanian women (Haj-Yahia 2002; Clark et al. 2009). Khawaja and Hammoury (2008) stated the factors associated with the acceptance of or justifying wife beating include young age, rural residence, agricultural work sector, low job status, low level of education, low household income and previous history of divorce or separation.

Yoshihama and Dabby (2015) stated that in violence against male partner, females commonly weren't the primary aggressors, but rather defending themselves and/or their children, or using revengeful violence.

The subjects in the current study were categorized into four groups; first was preschool group as schools offer a vehicle for social interaction, developing one’s identity, and building their self-esteem and confidence and may provide much needed support and protection (Mullender & Morley 1994). The second group was between school age (6 years) and the mean age of puberty (12 years), the third group was between the mean age of puberty and legal adulthood age to detect if risk of sexual type of abuse increase at adolescence age or not?, and the last group was adult age group.

The present study showed that there was a highly statistically significant difference between males and females regarding age groups (*p* = 0.000*), where females markedly outnumbered males in age groups from 12–18 years and age group 18 years and above. While the rate of victimization was equal in both sexes in earlier age groups (below 6 years and from 6 years to below 12 years).

Similar results were reported by Sinha (2012) (Sinha 2012) in Canada, where the rates of family violence were similar in boys and girls below three years of age, after that the rates started to diverge and the difference widened. These higher rates of family violence among girls and women, especially as they age, may be attributed to their much higher risk of sexual violence and presence of gender inequality in a patriarchal community.

The present study revealed that 75% of domestic violence victims reside rural houses while 19.5% of cases reside urban houses. The remaining 5.5% of cases live in shelters. This may reflect the importance of education level for a secure livelihood for the family as people live in rural and bedouin areas are mostly low educated or not educated at all (Al-Matalka 2009; Hocagil et al. 2016).

The highest percentage of studied cases (28.1%) occurred during the spring months, then they decreased through winter and summer months (25.8% and 25% respectively), to reach 21.1% % in autumn months.

There may be a relation between spring and aggravation of mood disorders, as manifested by increasing hospital admissions, mood disorder severity, electroconvulsive therapy use, and worsening of depression scores (Goodwin & Jamison 2007; Postolache et al. 2010). This could be explained by bioclimatic theory as seasonal variation in bright light, photoperiod, and environmental temperature, may induce adverse changes in neurotransmitter systems. Seasonal changes in neurotransmitter systems were previously reported by Maes et al. (1995) (Maes et al. 1995) where a precursor of serotonin; called plasma L-tryptophan (a neurotransmitter involved in depression pathophysiology); has a trough during spring months. Alternative biological factors have been proposed as triggers of violence in spring, such as the marked tree pollen peaks in spring that may cause seasonally increased cytokine production in respiratory tract resulting in exacerbation of mood disturbance in spring (Postolache et al. 2005; Guzman et al. 2007).

The current work reported that family and financial problems accounted for the 71.1% of all risk factors of domestic violence, followed by family disintegration (15.5%). This finding is congruent with the study carried out by Al-Matalka (2009) ; as family with low income or non-fixed income are more exposed to violence. Moreover, the study revealed other risk factors which are related to the victim; like studying or educational problems which represented 6.3% and mental problem (5.5%) or servants which accounted for 1.6% of cases. Violence is a highly complex phenomenon resulting from the interaction of many factors like biological, social, cultural, economic and political (Krug et al. 2002).

Unemployment rate accounted for 12.5% according to Jordan’s official department of statistics, but the rate is more than double that among people under 30, who represent nearly 70% of the population. On the other hand, education and literacy rates account for 90% of population which is considered high compared with other countries with similar incomes, but a gender gap still exists in illiteracy rates (females at 8.3% compared to 3.4% for males) (Department of Statistics (DOS 2013).

Araji and Carlson (2001) (Araji & Carlson 2001) reported that increased exposure of Jordanian to western culture through television, music, clothes and travel results in a conflict between traditional and modern ideas which may serve to increase the rate of family violence, especially to females.

Although Jordan has conservative Islamic values, drug abuse is increased and penetrated not only the capital city of Jordan; Amman, but also into villages and rural areas. As young people constitutes a significant proportion of Jordan’s together with increased use of nicotine, which is considered a gateway drug for alcohol, marijuana, and other drug abuse in addition to Jordan geographic location as lies in the path of drug exporters and buyers in Israel, Syria and Saudi Arabia (Haddad et al. 2010).

Regarding the type of violence, sexual abuse was the most common method of domestic violence in both genders (41.4%), followed by psychic and physical abuse (24.2%, 21.9% respectively). Psychic abuse was diagnosed and registered in the records after careful history taking, victim interrogation and then psychiatric examination of these cases. Victims may appear shy, with undue fear, stammering speech, depressed with loss of eye contact, poor behavioral control, low self-esteem, with personality and conduct disorders. Evidence of neglect was seen in 8.6% of cases as extensive dental caries, severe diaper dermatitis, neglected wound care, bald scalp areas related to severe malnutrition. Lastly mixed sexual and physical abuse accounted for 3.9% of cases. While in a study in Irbid city and Balka (in north Jordan) psychic abuse had the highest prevalence among all types of abuse (Al-Nsour et al. 2009). This may be explained by higher education, better financial condition or more civilization at north.

Sexual violence has a profound impact on the physical, mental, sexual and reproductive health of the victims. These consequences are seen both immediately and many years after the assault. Mortality associated with sexual violence may occur through suicide, HIV infection, unsafe abortion and murder, either during the attack or subsequently in “honor killings” crimes (Krug et al. 2002; Akinlusi et al. 2014).

The physical abuse was the commonest type of domestic violence committed in the young age groups (below 6y and from 6 to 12 years), while the incidence of sexual abuse assaults increased with increasing age, where sexual abuse accounted for the highest percentage of abuse in older age groups (from 12 to 18 years and ≥ 18 years). None of cases of neglect occurred in age group of 18 years and above. There was a highly statistical significant association between age group of the studied cases and the type of violence, where P = 0.000*.

Similarly, Kirschner and Wilson (2001) (Kirschner & Wilson 2001) reported that young children were most at risk of physical abuse, whereas the highest rates of sexual abuse were among children who have reached puberty or adolescence. Mostly, boys are the victims of physical punishment and beating more often than girls, while girls are at higher risk of infanticide, sexual abuse and neglect (Hunter et al. 2000). This also coincides with other studies in Egypt and Palestine, where the highest percentage of sexual abuse cases was distributed across middle childhood and early adolescence (Aboul-Hagag & Hamed 2012; Elgendy & Hassan 2013; Haj-Yahia & Tamish 2001).

The present study revealed a highly statistical significant relation between gender and the type of domestic violence. Physical abuse represented the commonest type of abuse committed in males (58.6%), while sexual abuse was the most frequent type of abuse committed in females (45.4%) followed by psychic abuse (30.3%). Moreover, only one case of psychic abuse was reported in males. Females were more than six times as likely as males to be a victim of sexual assault (6.25:1) either as a sole assault (45.4%) or mixed with physical one (5%).

Similarly, females in Canada were more than four times as likely as boys to be a victim of sexual assault or other sexual offences committed by a family member (Sinha 2012).

The present study revealed a highly statistical significant relation between risk factor and the type of domestic violence. All mentally retarded cases were abused physically and the two servant cases were sexually abused as this vulnerable population has no ability to leave abusive relationships. Mental health problems might be a risk factor for repetitive violence as described by Barnett (2001). The current study showed that nearly half of cases of family disintegration became sexually abused victims which may be due to lack of adequate supervision.

The present study revealed that leaving home was the commonest outcome of domestic violence due to constant anxiety, gloomy future, hopelessness, pessimism, sense of insecurity, and lack of goal in life. Actually domestic violence negatively affects all members of the family who are brought up in hostility and increases the chances of divorce, broken homes and other social problems. (Al-Matalka 2009)

Nearly one fifth of cases had hymenal tears i.e. lost their virginity and another 12.5% had illegal pregnancy as a consequence of sexual abuse including incest. Anal tears accounted for 10% of overall outcome, while a case of combined hymenal and anal tears was reported in a female victim aged 15.5 years old. These findings were very serious as crimes of honor in Jordan appear to be a significant percentage of total female homicides; and this may be attributed to reduction of penalty of assailants committing crimes of honor in Jordanian law (Hadidi et al. 2001).

The time interval between sexual assaults and disclosure varied widely from less than 24 h to six months (24 weeks pregnancy). This wide variation in the interval of disclosure may be due to threats or fear of stigmatization, violence or even death. The longer the interval between assault and its disclosure, the lower the quantity and quality of forensic evidences, also the higher the risk of negative health outcomes (World Health Organization 2003).

There was a highly statistical significant relation between age group and outcome. The highest percentage of skin scars (45.5%) was reported among those with age group from 6 to 12 years. On contrary, the highest percentages of hymenal tears and leaving home outcomes were recorded in older age group 18 years and above (65.4% and 56.6% respectively).

There was a highly statistical significant relation between gender and outcome. The highest percentage of skin scars occurred among males (72.7%) as they are more liable to physical assaults, while all cases of leaving home and 81.8% of cases with speech and psychic disorders were females as they are more sensitive than males.

There was a highly statistical significant relation between type of violence and outcome. It was recorded that 78.5% of physically abused victims developed skin scars and 80.6% of psychic abuse cases leaving their home as an outcome. Regarding sexually abused victims, it was recorded that 41.5% of them had hymenal tears and 30.2% of them had illegal pregnancy. It is worth noting that none of victims died as a consequence of their abuse and this denotes that the abuses were not severe enough to cause death.

## Conclusion

Conclusively, the study highlighted the amplitude of problem of domestic violence in south region of Jordan. Also, it assessed victims’ characteristics, types of assaults with their outcome and consequences. The study showed the risk for sexual victimization among adolescents and young girls with a high percentage of post-assault conception.

### Recommendations

Violence is not inevitable problem and we can do much to prevent it through improving parenting skills; promoting awareness among people about the negative impact of violence on a family. We also should stress the principle of gender equality and the need for safeguarding and providing a secure livelihood for the family through effective prevention strategies performed by government and non-governmental organization services, with enforcement of law legal punishment against domestic violence and honor crimes. The study also recommended more researches to investigate the problems and the cultural effects on violence particularly in rural and Bedouin areas. As the study faced many difficulties regarding data registration in medico-legal reports, a more specific comprehensive scheme is recommended, including data about the offenders and their relation to victims together with the need to address substance use, unemployment and other risk factors of violence among Jordanians to make the process of registration more accurate and convenient.
